# Successful heart transplantation from a COVID‐19‐positive donor: A clinical case report

**DOI:** 10.1002/ccr3.8564

**Published:** 2024-03-04

**Authors:** Mohsen Meidani, Maryam Moradi, Fereshteh Ghiasvand, Neda Alijani

**Affiliations:** ^1^ Department of Infectious Diseases, School of Medicine, Imam Khomeini Hospital Complex, School of Medicine Tehran University of Medical Sciences Tehran Iran; ^2^ Eye Research Center, The Five Senses Health Institute, Rassoul Akram Hospital Iran University of Medical Sciences Tehran Iran; ^3^ Department of Infectious Diseases, Liver Transplantation Research Center, Imam Khomeini Hospital Complex, School of Medicine Tehran University of Medical Sciences Tehran Iran; ^4^ Department of Infectious Disease , Dr. Shariati Hospital, School of Medicine Tehran University of Medical Sciences Tehran Iran

**Keywords:** COVID‐19, heart transplantation, positive COVID‐19 organ donor, transplantation

## Abstract

**Key Clinical Message:**

Despite the acceptable results in patients receiving organs from COVID‐19‐positive donors, more extensive studies over a longer period are still needed for more accurate conclusions.

**Abstract:**

Organ transplantation is a major concern during the current COVID‐19 pandemic worldwide. The use of COVID‐19‐positive organ donors has raised widespread concerns in the field of transplantation. In this study, we characterized the outcome of a heart transplant from an organ donor positive for COVID‐19.

## 1 INTRODUCTION

During any pandemic, several crises have appeared with an additional need for various studies and approved guidelines to take action. Heart and other transplantations have become important issues worldwide during the recent COVID‐19 pandemic. The use of COVID‐19‐positive organ donors has raised widespread concerns in the field of transplantation.^1^

Various studies have demonstrated that heart transplant recipients receiving positive COVID‐19 donor organs have reassuring short‐term outcomes with adequate screening and prophylactic treatments.^2^, ^3^

In this study, we characterize the outcome of a heart transplant from an organ donor, positive for COVID‐19.

## 2 CASE HISTORY/EXAMINATION

A 37‐year‐old female with a history of heart transplantation was admitted to Imam Khomeini Hospital, Tehran, Iran, in September 2023 because of heart failure. In the 3 months leading to her presentation, she developed mild dyspnea and chest tightness on exertion. The patient had no preceding viral illnesses.

**FIGURE 1:**
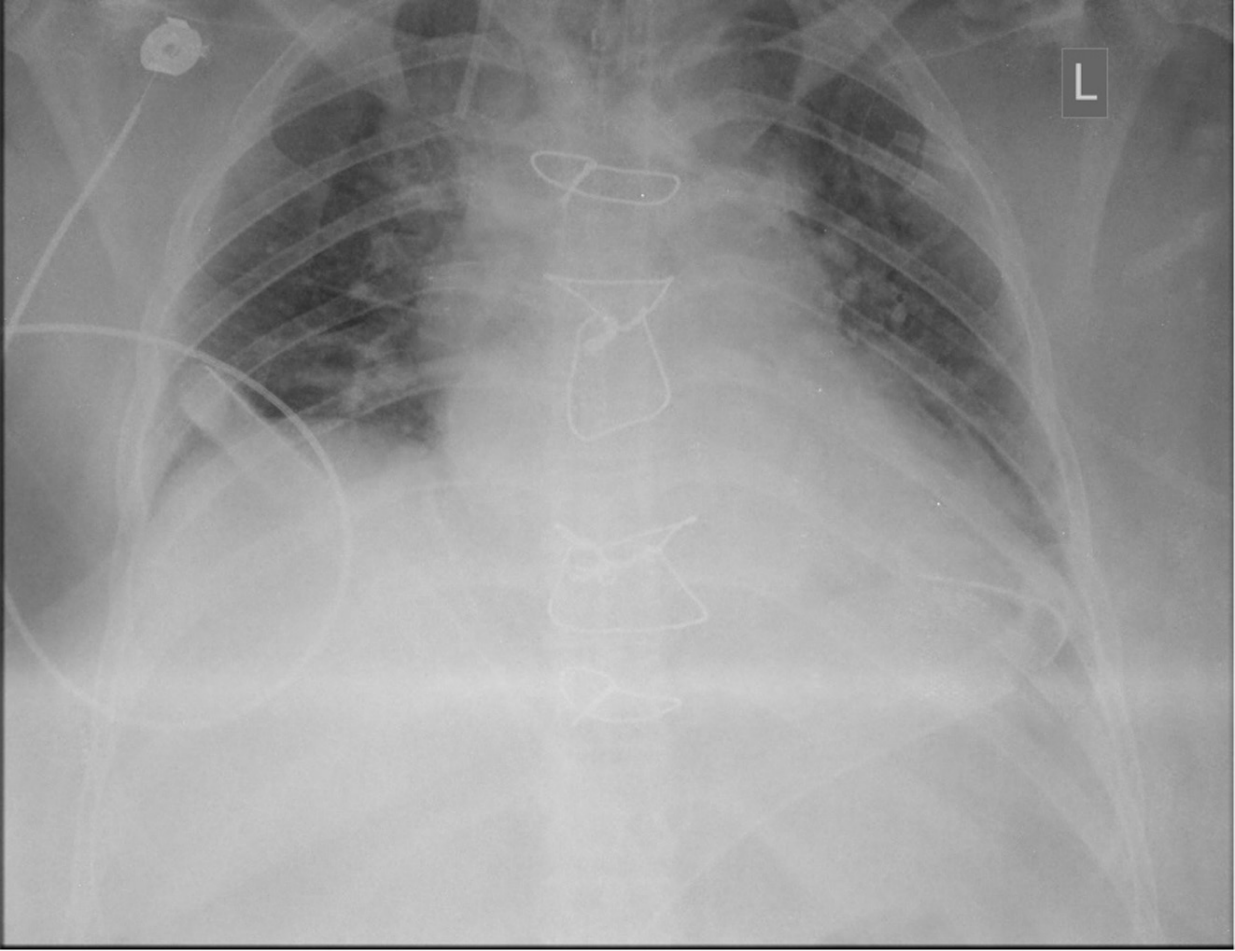
CXR; Mild cardiomegaly and mild enlargement of the hilar pulmonary vessels.

In the emergency department, her blood pressure was 120/80 mmHg, heart rate was 90 beats per min, respiratory rate was 14 breaths per min, and oxygen saturation was 98% while breathing room air and the temperature was 36.9°C. Clinical examination was unremarkable for jugular venous distension or heart murmurs. Laboratory analysis results were all within the normal ranges. Chest radiography (CXR) revealed mild cardiomegaly and mild enlargement of the hilar pulmonary vessels. (Figure 1). Electrocardiogram revealed sinus tachycardia and ST elevations V2–V4, and transthoracic echocardiography revealed a dilated left ventricle with an ejection fraction of approximately 10%.

She underwent cardiac transplantation in 2010 for the first time because of an unknown etiology of heart failure. Subsequently, she underwent percutaneous coronary intervention with stent insertion in 2013 for coronary artery disease requiring acute management. Then, she required permanent pacemaker insertion in 2021 due to the development of heart failure. On admission, cardiac retransplantation was decided based on the patient's clinical condition and echocardiographic findings.

## 3 METHODS

After infection screening, she was administered 125 mg of methylprednisolone, 1 mg of tacrolimus, and 500 mg of CellCept every 12 h. For infection prophylaxis, co‐trimoxazole 400/80 mg and valganciclovir 450 mg twice daily were prescribed.

**FIGURE 2:**
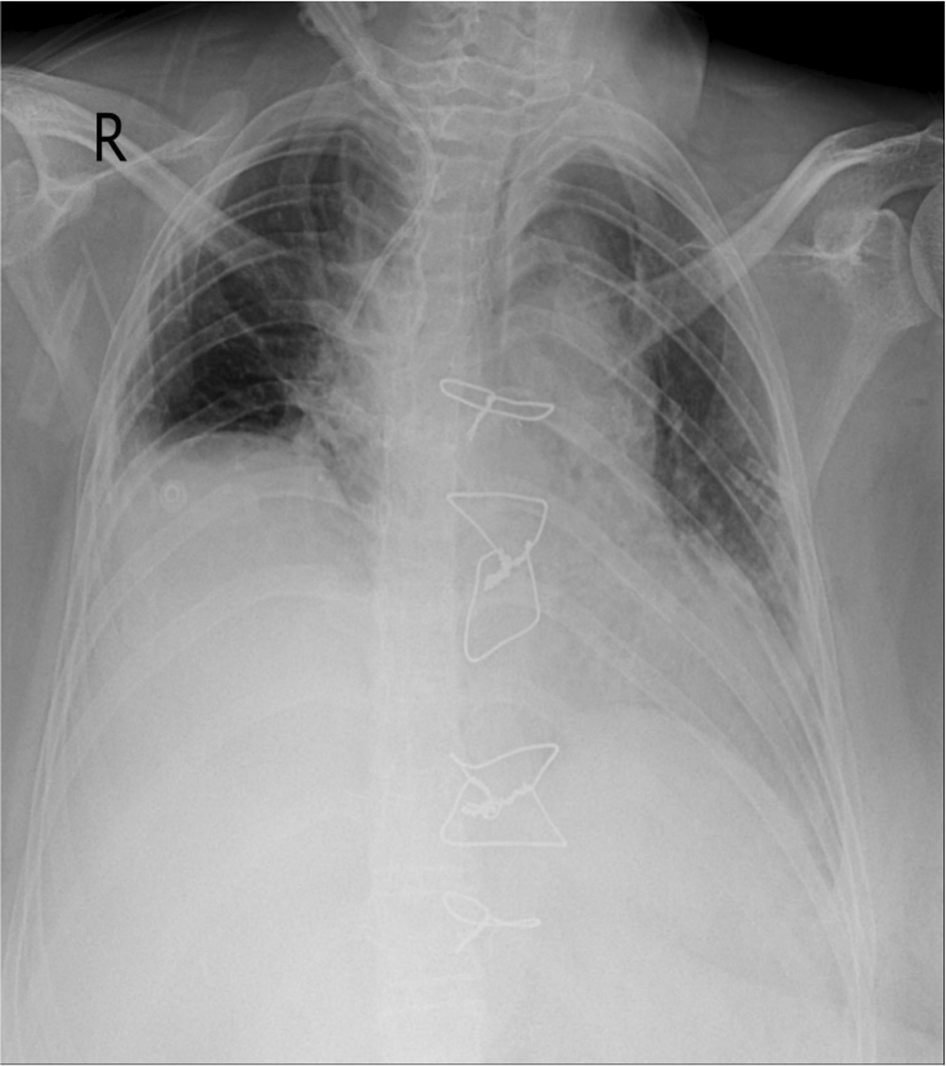
Bilateral central consolidation in favor of aspiration pneumonia without significant evidence of COVID‐19.

She underwent a heart transplant from a brain‐dead 20‐year‐old male with a positive COVID‐19 polymerase chain reaction (PCR) test. Postoperative echocardiography revealed a 55% ejection fraction with acceptable valve motion. On day 4 after the transplant, rewiring and on day 6, tracheostomy was performed because of prolonged intubation due to ventilator‐associated pneumonia. On CXR, bilateral central consolidation was detected in favor of aspiration pneumonia (Figure 2). The patient was treated with meropenem (2 g every 8 h intravenously), amikacin (1 g/day), and vancomycin (1 g) and had a favorable clinical response.

## 4 CONCLUSION AND RESULTS

Fortunately, postsurgical PCR for COVID‐19 was negative twice in the recipient, unless the donor had positive COVID‐19 PCR results. The patient was discharged under acceptable clinical conditions and scheduled follow‐up sessions.

Considering the shortage of heart donors, organ donation from COVID‐19‐positive patients is safe and does not pose a risk of ARDS or other complications in transplant recipients. Therefore, accepting organs from these patients can save their lives on the waiting list.

## 5 DISCUSSION

Even after the World Health Organization (WHO) declaration about the end of the COVID‐19 pandemic, new variants are still emerging. According to previous studies, SARS–CoV–2‐positive donor hearts are known to increase the number of available donor hearts, reduce waiting periods, and improve survival rates for patients waiting for heart transplants.^4^, ^5^ Based on recent updates in Recommendations and Guidance for Organ Donor Testing and Evaluation, non‐lung donors are accepted as heart donors because of the low possibility of transmission of infection.^6^

Samuel T. Kim and his colleagues declared that there was no significant difference between in‐hospital, 30‐day, and cumulative death in positive COVID‐19 and non‐positive COVID‐19 heart donors. Postsurgical complications including, infections, graft rejection, multi‐organ failure, stroke, and dialysis were not higher in those received from positive COVID‐19 heart donors.^3^ Duration of hospitalization and the overall outcome, including 30‐day and 6‐month survival were also unaffected in patients with COVID‐19‐positive heart donors.^7^

Unlike the majority of studies, Madan et al. demonstrated that the 6‐month and 1‐year mortality rates were higher in patients with COVID‐19‐positive heart donors than in those with COVID‐19‐negative heart donors.^8^

Similar to other case presentations, our patient was discharged with a good clinical outcome after a SARS–CoV–2‐positive donor heart transplant with a negative postsurgical COVID‐19 PCR test.

Based on our experience, previous studies, and the American Heart Association declaration, successful heart transplantation from donors with positive PCR for COVID‐19 is expected.

Despite the acceptable results in patients receiving organs from COVID‐19‐positive donors, more extensive studies over a longer period are needed for more accurate conclusions.

## AUTHOR CONTRIBUTIONS

**Mohsen Meidani:** Project administration; validation. **Maryam Moradi:** Writing – original draft. **Fereshteh Ghiasvand:** Validation. **Neda Alijani:** Resources; supervision.

## CONSENT

Written informed consent was obtained from the patient to publish this report in accordance with the journal's patient consent policy.

## Data Availability

Data sharing does not apply to this article as no datasets were generated or analyzed during this study.
